# Low Grade Lymphoma Mimicking Metastatic Urothelial Carcinoma: When Do We Need Further Histologic Staging?

**DOI:** 10.1155/2016/8125898

**Published:** 2016-11-22

**Authors:** Azka Ali, William P. Skelton IV, Neeka N. Akhavan, Thu-Cuc Nguyen, Zachary A. Taylor, Tabitha Townsend, Prajwol Pathak, Nalini Hasija, Li Li, Jacqueline Indrisek, Scott Watson, Isis Nixon, Nam H. Dang, Robert Zlotecki, Paul Crispen, Robert Allan, Patricia Abbitt, Long H. Dang

**Affiliations:** ^1^Department of Internal Medicine, University of Florida, Gainesville, FL, USA; ^2^Department of Internal Medicine, University of Central Florida, Orlando, FL, USA; ^3^Division of Hematology and Oncology, Department of Internal Medicine, University of Florida, Gainesville, FL, USA; ^4^Department of Radiation Oncology, University of Florida, Gainesville, FL, USA; ^5^Department of Urology, University of Florida, Gainesville, FL, USA; ^6^Department of Pathology, University of Florida, Gainesville, FL, USA; ^7^Department of Radiology, University of Florida, Gainesville, FL, USA

## Abstract

*Introduction.* Patients with urothelial carcinoma of the bladder often present with metastases to regional lymph nodes, with lymphadenopathy on physical examination or radiographic imaging.* Case Presentation.* We present the case of a 73-year-old Caucasian man with presumed metastatic urothelial carcinoma of the bladder to regional pelvic and retroperitoneal lymph nodes. He underwent systemic chemotherapy for treatment of urothelial carcinoma and was discovered on restaging to have findings suggestive of disease progression but ultimately was found to have a concurrent secondary malignancy.* Conclusion.* Our case suggests that in patients with urothelial carcinoma, the concurrent presentation of regional lymphadenopathy may not be metastatic urothelial carcinoma and may warrant further investigation.

## 1. Introduction

Each year, 74,000 people are diagnosed with urothelial carcinoma of the bladder in the United States [1]. Of this group, one-third will have muscle-invasive disease on presentation, and half of those patients will have lymph node involvement or distant metastases [2]. Urothelial bladder carcinoma originates in the bladder mucosa, subsequently spreading to the lamina propria, muscularis propria, perivesical fat, nearby pelvic structures, and ultimately to the lymph nodes marking progression of the disease [3]. Untreated, muscle-invasive bladder cancer has a two-year mortality approaching 85% [4]. The most significant factors in determining survival in bladder cancer are primary tumor stage and lymph node metastasis; metastases are staged as N1, N2, or N3 according to the TNM system based on the number and size of metastatic nodes [5].

The gold standard therapy for high grade muscle-invasive urothelial carcinomas is neoadjuvant chemotherapy followed by radical cystectomy with urinary diversion [4]. Prior to the 1990s, radical cystectomy alone was the standard therapy. Multiple randomized controlled trials in the 1990s and 2000s led us to determine a substantial advantage of neoadjuvant chemotherapy in improving bladder cancer related mortality [6]. The current neoadjuvant chemotherapy standard is combined with MVAC (methotrexate, vinblastine, doxorubicin, and cisplatin) or GC (gemcitabine and cisplatin) [6]. Another acceptable curative treatment option is TURBT followed by definitive chemoradiation [7].

A second primary cancer (SPC) is defined by the National Cancer Institute, as a new primary malignancy that occurs in a patient with a prior history of cancer [8]. There is very little information in the literature, regarding second primary cancers in the setting of known urothelial carcinoma of the bladder. This case describes a patient with the diagnosis of urothelial carcinoma with lymph node spread, who on subsequent biopsy of lymph nodes was found to have a second primary B cell lymphoma.

## 2. Case Presentation

**Figure 1:**
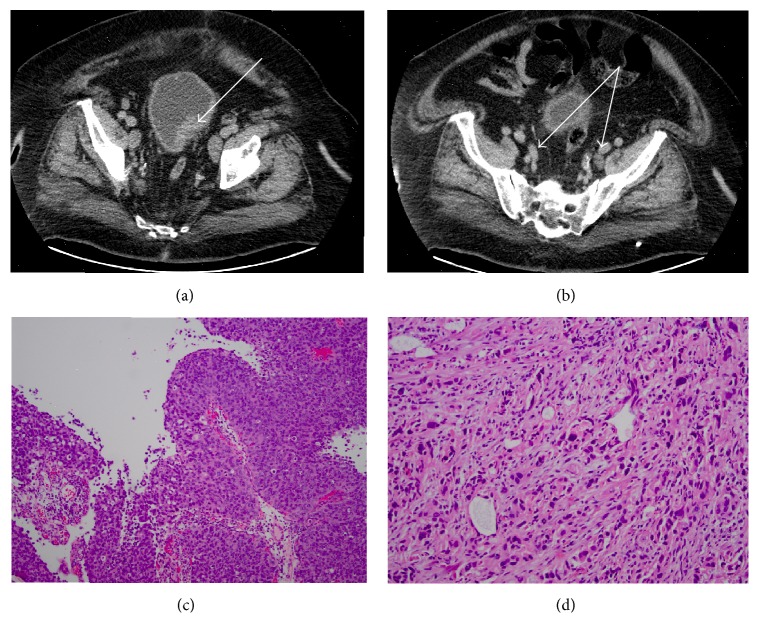
(a) Bladder mass, before chemotherapy. (b) Pelvic lymphadenopathy, before chemotherapy. (c) 100x: biopsy of bladder, before chemotherapy, and surface papillary component of the tumor. (d) 200x: biopsy of bladder, before chemotherapy; shown here are invasive malignant cells.

The patient presented here is a 73-year-old Caucasian man with presumed metastatic urothelial carcinoma of the bladder. He had a history of congestive heart failure, hypertension, obstructive sleep apnea, and morbid obesity (BMI 50). His past surgical history included appendectomy, bilateral hip replacement, and pacemaker placement. Urothelial carcinoma was initially discovered by computed tomography (CT) scan of the abdomen and pelvis, which was performed as a workup of persistent abdominal pain, anorexia, and weight loss. The CT scan showed large posterior-lateral dome bladder thickening that measured 4.6 cm × 2.5 cm, 3 mm lung nodule, bilateral exophytic hypodensities of the kidneys, and pelvic and retroperitoneal lymphadenopathy (Figures 1(a) and 1(b)).

**Figure 2:**
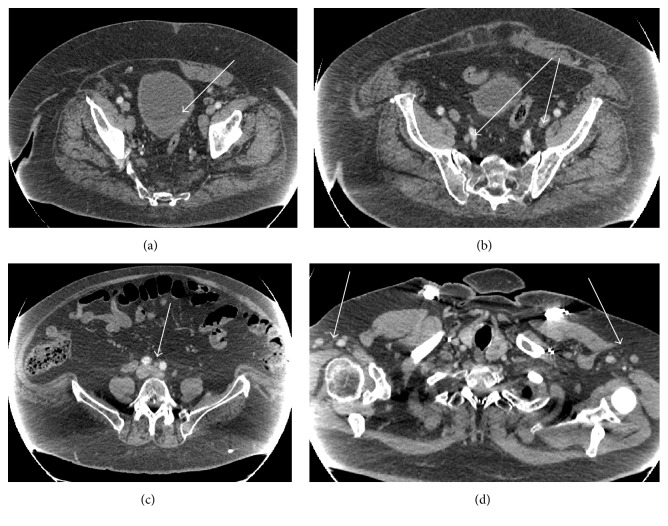
(a) Bladder mass, after chemotherapy. (b) Pelvic lymphadenopathy, after chemotherapy. (c) Retroperitoneal lymph node above aortic bifurcation, after chemotherapy. (d) Axillary lymph nodes, after chemotherapy.

Cystoscopy revealed a large bladder mass. The patient underwent transurethral resection of bladder with complete excision of mass, which involved one-third of the bladder and weighed 23 grams. Pathology confirmed high grade invasive urothelial carcinoma of the bladder with indeterminate lymphovascular invasion and was staged as T2N3M1 (Figures 1(c) and 1(d)). Due to the extent of lymph node involvement which likely represented metastatic urothelial cancer, the patient was not deemed an appropriate surgical candidate. He was started on GC combination chemotherapy with the goal of curative surgery or, if lymph nodes persisted, would continue to definitive chemoradiation. The treatment course was complicated with an episode of urinary retention and urinary tract infection. He developed thrombocytopenia with platelet count drop from 185,000 to 63,000, and subsequently, day 15 of cycle 1 chemotherapy was held. Initially, GC frequency was reduced from three weekly doses every 28 days to every other week dosing, and then gemcitabine was dose-reduced by 20%. After 3 months of chemotherapy, follow-up CT scan showed further progression of lymphadenopathy with prominent lymph nodes in axilla (one on the left measuring 17 mm and one on the right measuring 13 mm), mediastinal and hilar nodes 9–11 mm in short axis, retroperitoneal nodes (prominent node measuring 25 × 13 mm above the aortic bifurcation on the right), and interval growth in external iliac and pelvic nodes (Figures 2(a)–2(d)).

**Figure 3:**
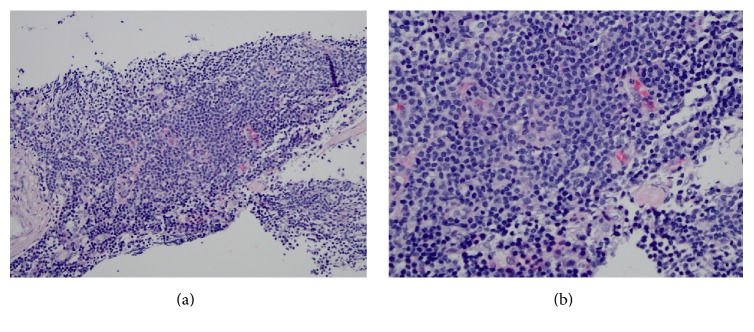
(a) 200x: left axillary monotonous population of small lymphocytes with mature nuclear chromatin and distinct rims of cytoplasm. (b) 400x: left axillary monotonous population of small lymphocytes with mature nuclear chromatin and distinct rims of cytoplasm.

**Figure 4:**
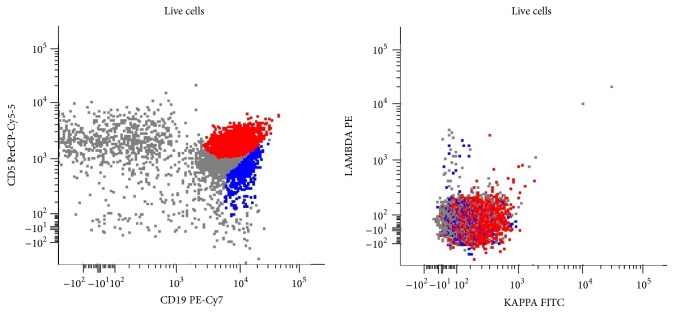
Flow cytometry of core needle biopsy: surface kappa clonal and CD19/CD20+ B cell population along with coexpression of CD5 and CD23 (tumor = red population; background polyclonal B cells = blue).

He underwent core biopsy of left axillary and right iliac nodes, which revealed low grade B cell lymphoma. Histologic sections of the axillary node showed a monotonous population of small lymphocytes with mature nuclear chromatin and distinct rims of cytoplasm (Figures 3(a) and 3(b)). Flow cytometric studies from this specimen showed a dim surface kappa clonal, relatively bright CD19/CD20+ B cell population with coexpression of CD5 and CD23 without expression of CD10 or significant CD38 (Figure 4). Additional immunohistochemistry showed expression of MUM1 without expression of BCL6, Cyclin D1, or SOX11. The differential diagnosis of this low grade B cell lymphoma included marginal zone lymphoma and lymphoplasmacytic lymphoma and less likely small lymphocytic lymphoma. The biopsy of the inguinal lymph node showed similar morphologic and immunophenotypic findings (not shown here). Neither contained evidence of metastatic urothelial carcinoma. Repeat bladder wash revealed normal bladder cells. There was no indication for treatment of low grade B cell lymphoma. Radiation oncology and medical oncology chose to continue with definitive concurrent cisplatin and radiation therapy for curative intent for his urothelial carcinoma of the bladder, which was restaged as T2N0M0.

## 3. Discussion

In this case, the patient was diagnosed with urothelial carcinoma, initially shown on CT scan of abdomen and pelvis, which showed thickening of the posterior-lateral dome of the bladder measuring 4.6 cm × 2.5 cm, as well as significant pelvic and retroperitoneal lymphadenopathy. After pathology confirmed invasive high grade papillary urothelial (transitional cell) carcinoma with muscularis propria involvement, this cancer was staged as T2N3M1.

Repeat imaging was conducted after appropriate treatment of 3 months of GC combination chemotherapy, which showed the presence of multiple lymph nodes, included in the left axilla, right axilla, retroperitoneum, external iliac, and pelvis. At that time, the differential diagnosis included persistent and progressive metastatic involvement with urothelial carcinoma, a lymphoproliferative disorder, or a secondary carcinoma of unknown primary. There was a high suspicion that the persistent lymphadenopathy was not due to metastatic involvement of the urothelial carcinoma, due to the atypical spread to axillary nodes. To distinguish the aforementioned etiologies, core needle biopsies of the left axillary lymph node and right iliac lymph node were performed, which both showed low grade B cell lymphoma. No metastatic carcinoma was detected in the biopsy of either lymph node, suggesting that the lymphadenopathy was due to a second primary malignancy rather than metastatic spread of the urothelial carcinoma. Therefore, based on the fact that low grade B cell lymphoma was biopsy proven in the lymph nodes, the cancer was appropriately restaged as T2N0M0. The patient is now treated for curative intent for his urothelial carcinoma with concurrent chemoradiation.

A second primary malignancy is defined as a cancer that has “arisen independently and not as a result of resurgence, nor as a result of metastasis of the original primary cancer [9].” Oftentimes, many second primary malignancies arise after treatment with radiation or chemotherapy. Radiation exposure is most commonly associated with the development of leukemia, thyroid, or female breast cancer [10], although it has also been associated with development of lung, stomach, esophagus, colon, bladder, ovarian, brain/CNS, and liver cancer [10].

Chemotherapies, including alkylating agents [11], cisplatin [12], and topoisomerase II inhibitors [13], can also increase the risk of the development of second primary cancers. These are most often associated with the development of leukemia and myelodysplastic syndromes.

There are sparse reports of second primary malignancies in the setting of urothelial carcinoma of the bladder. These include reports of urothelial carcinoma developing synchronously with esophageal squamous cell carcinoma [14], urothelial carcinoma presenting with malignant Brenner tumor [15], and prostatic and urothelial carcinoma metastases detected in the same lymph node [16].

In addition to the synchronously detected second primary cancers, there are also reports of invasive bladder cancer developing after inverted papilloma [17, 18]. Transitional cell carcinoma has also been reported after treatment of lymphoma with cyclophosphamide [19]. There is also evidence of lower tract urothelial carcinoma developing in patients with prior upper tract urothelial carcinoma [20]. Urothelial carcinoma has also been reported prior to the development of thyroid cancer, with some evidence that this may be a rare sporadic neoplastic syndrome [21]. There has also been a report of urothelial carcinoma with a second cancer where it was found that the second cancer (in this case, breast cancer) was in fact metastatic spread of the urothelial carcinoma [22].

Therefore, with respect to our case, it is one of the rare cases of a second primary cancer associated with urothelial carcinoma. Our case is the first presentation of concurrent urothelial carcinoma and lymphoma, confounding clinical staging and treatment decision. On initial assessment, the lymphadenopathy was attributed to metastatic spread of the urothelial carcinoma, which is the most common clinical presentation. The second primary B cell lymphoma was only detected after the atypical progression of lymphadenopathy. Although the lymphadenopathy visible on the initial CT scan was almost assuredly a second primary B cell lymphoma, it is impossible to definitively state as there was no lymph node biopsy performed at the time of diagnosis. It is extremely unlikely that the lymphoma developed as a result of cisplatin chemotherapy due to the short duration of only three months of therapy prior to discovery of second primary B cell lymphoma.

## 4. Conclusion

Our study is the first to show that urothelial carcinoma and lymphoma may present at the same time, which may complicate clinical staging and treatment decisions. For urothelial carcinoma, in cases of persistent or progressive lymphadenopathy despite appropriate systemic treatment, it is important to consider the possibility of a second primary malignancy and adjust treatment upon further histologic staging.

## References

[1] Siegel R. L., Miller K. D., Jemal A. (2016). Cancer statistics, 2016. *CA: A Cancer Journal for Clinicians*.

[2] Tonyali S., Yazici S. (2016). Does solitary- and organ-confined metastasectomy really improve survival in advanced urologic malignancies?. *International Urology and Nephrology*.

[3] Stein J. P., Lieskovsky G., Cote R. (2001). Radical cystectomy in the treatment of invasive bladder cancer: long-term results in 1,054 patients. *Journal of Clinical Oncology*.

[4] Cahn D. B., Ristau B. T., Ghiraldi E. M. (2016). Bladder preservation therapy: a review of the literature and future directions. *Urology*.

[5] Liedberg F., Månsson W. (2006). Lymph node metastasis in bladder cancer. *European Urology*.

[6] Winquist E., Kirchner T. S., Segal R., Chin J., Lukka H. (2004). Neoadjuvant chemotherapy for transitional cell carcinoma of the bladder: a systematic review and meta-analysis. *Journal of Urology*.

[7] Ibrahim S. M., Abd El-Hafeez Z. M., Mohamed E. M., Elsharawy I. A., Kamal K. M. (2007). Transurethral Resection of Bladder Tumor (TUR-BT) then concomitant radiation and cisplatin followed by adjuvant gemcitabine and cisplatin in muscle invasive Transitional Cell Carcinoma (TCC) of the urinary bladder. *Journal of the Egyptian National Cancer Institute*.

[8] Chakraborty S., Tarantolo S. R., Batra S. K., Hauke R. J. (2013). Incidence and prognostic significance of second primary cancers in renal cell carcinoma. *American Journal of Clinical Oncology*.

[9] Feller L., Lemmer J. (2012). New ‘second primary’ cancers. *SADJ: Journal of the South African Dental Association*.

[10] Travis L. B. (2006). The epidemiology of second primary cancers. *Cancer Epidemiology Biomarkers and Prevention*.

[11] Radford J., Longo D. L. (2015). Second cancers after treatment for Hodgkin's lymphoma—continuing cause for concern. *The New England Journal of Medicine*.

[12] Dertinger S. D., Avlasevich S. L., Torous D. K. (2014). Persistence of cisplatin-induced mutagenicity in hematopoietic stem cells: implications for secondary cancer risk following chemotherapy. *Toxicological Sciences*.

[13] Pendleton M., Lindsey R. H., Felix C. A., Grimwade D., Osheroff N. (2014). Topoisomerase II and leukemia. *Annals of the New York Academy of Sciences*.

[14] Vilcea I. D., Vasile I., Tomescu P. (2010). Synchronous squamous esophageal carcinoma and urothelial renal cancer. *Chirurgia*.

[15] Bouda J., Hes O. (1999). An unusual case of malignant Brenner tumor in association with low-grade urothelial carcinoma of the urinary bladder. A case report. *European Journal of Gynaecological Oncology*.

[16] Pacella E., Ricci F., Colecchia M., Boccardo F., Lopez-Beltran A., Spina B. (2015). Prostatic and urothelial metastasis in the same lymph node: a case report. *Analytical and Quantitative Cytology and Histology*.

[17] Koizumi K., Shiga J., Yokota H. (2013). A case of inverted papilloma of the renal pelvis, associated with metachronous urothelial carcinoma of the urinary bladder. *Acta Urologica Japonica*.

[18] de Knijff D. W., Theunissen P. H., Delaere K. P. (1997). Inverted papilloma of the ureter with subsequent invasive bladder cancer. *Acta Urologica Belgica*.

[19] Shiga Y., Suzuki K., Tsutsumi M., Ishikawa S. (2002). Transitional cell carcinoma of the renal pelvis in a patient with cyclophosphamide therapy for malignant lymphoma: a case report and literature review. *Acta Urologica Japonica*.

[20] Kates M., Badalato G. M., Gupta M., McKiernan J. M. (2012). Secondary bladder cancer after upper tract urothelial carcinoma in the US population. *BJU International*.

[21] Albores-Saavedra J., Dorantes-Heredia R., Chablé-Montero F., Córdova-Ramón J. C., Henson D. E. (2014). Association of urothelial carcinoma of the renal pelvis with papillary and medullary thyroid carcinomas. A new sporadic neoplastic syndrome?. *Annals of Diagnostic Pathology*.

[22] Dumont C., Gauthier H., Vérine J. (2014). Concomitant bifocal urothelial carcinoma and breast tumor: second primary cancer or metastatic spread to the breast?. *Case Reports in Oncological Medicine*.

